# Tea Catechins: Potential Plant-Derived Feed Additives for Improving Chicken Intestinal Health and Productivity

**DOI:** 10.3390/ani15111553

**Published:** 2025-05-26

**Authors:** Bing Tian, Wenjing Zhuang, Yanle Fan, Yun Hu, Xiaoyan Cui, Tingting Li, Liyang Zhang, Xugang Luo, Shengchen Wang

**Affiliations:** 1 College of Animal Science and Technology, Yangzhou University, Yangzhou 225000, China; bingtian0201@163.com (B.T.); 17856855846@163.com (W.Z.); fanyl0303@163.com (Y.F.); huyun@yzu.edu.cn (Y.H.); xycui@yzu.edu.cn (X.C.); di50070@yzu.edu.cn (T.L.); 2 State Key Laboratory of Animal Nutrition, Mineral Nutrition Research Division, Institute of Animal Science, Chinese Academy of Agricultural Sciences, Beijing 100193, China; zhangliyang@caas.cn

**Keywords:** catechins, chicken, feed additives, antioxidant function, gut microbiota

## Abstract

In response to the global antimicrobial resistance and residue issues, most countries and regions have implemented comprehensive bans on the use of growth-promoting antibiotics in animal feed formulations. Catechins are a group of polyphenolic compounds that are naturally present in many fruits and plant-derived foods, emerging as potential antibiotic-alternative additives for promoting gut health and production performance in poultry farming. The compounds demonstrate dual biological functions: mitigating oxidative tissue damage and actively remodeling gut microbiota composition by suppressing enteric pathogens (e.g., *Clostridium*) and enriching beneficial bacteria (e.g., *Bifidobacterium*). Such multifaceted regulation enhances intestinal barrier integrity, thereby improving nutrient absorption and translating into measurable production benefits, including elevated growth rates and feed conversion efficiency. The current research suggests that the added levels of catechins in chicken diets ranges from 40 to 600 mg/kg. However, the research and application of catechins in chicken production are still at the initial stage, which requires more attention worldwide and further mechanism exploration. This mini-review synthesizes recent research findings on the effects of catechins on chicken health, aiming to promote the formulation of feed additive strategies using plant-derived compounds such as catechins as substitutes for antibiotics, to maintain the health of poultry and other animals while preserving production performance.

## Simple Summary

Catechins, natural polyphenols, show promise as antibiotic alternatives in poultry feed by improving gut health, reducing oxidative stress and balancing microbiota. However, the research on catechins in poultry is nascent, with unclear mechanisms. This review supports catechins as sustainable feed additives to replace antibiotics as a new growth-promoting factor in maintaining chicken intestinal health and productivity.

## 1. Introduction

Since the last century, low-dose antibiotics have been widely used as growth promoters in the breeding industry to maintain animal health and improve feed conversion efficiency by inhibiting or killing pathogenic microorganisms and improving tissue metabolism [1]. However, despite the significant benefits brought by the use of antibiotics, they also pose serious threats to public health [2,3]. Currently, the global consumption of antibiotics in livestock and poultry is nearly twice that of humans. Therefore, the raising of livestock and poultry is considered a major source of antibiotic emissions and bacterial resistance [4]. For instance, antibiotics such as amphenicol and tetracycline have been detected in food products, posing risks to consumer health [5]. Hur et al. reported that intestinal *Salmonella* strains isolated from eggs and chicken carcasses are resistant to a variety of widely used antibiotics, such as penicillin, streptomycin, tetracycline, sulfamethoxazole, and quinolones [6]. Given the increasing concerns about antibiotic resistance and residue issues, since 2006, many countries and regions worldwide have completely banned the addition of antibiotics as growth factors to diets [7,8]. Consequently, the development and research of safe and effective alternatives to feed antibiotics have emerged as a major focus. Recently, phytogenic bioactive compounds such as polysaccharides, flavonoids, saponins, and other compounds have been found to have antioxidant, anti-inflammatory, growth-promoting, and gut health-enhancing effects [9,10]. Unlike traditional antibiotics that broadly suppress gut microbiota, plant-derived bioactive substances promote livestock and poultry health through gut microbiota remodeling and comprehensive protective mechanisms. Moreover, some of these components can effectively inhibit some antibiotic-resistant strains, highlighting the effectiveness of their microbial effects [11,12]. This shift emphasizes the inherent advantages of phytogenic bioactive compounds in developing feed-grade alternative products and their development needs in line with natural metabolic strategies to maintain animal health and production performance.

As a type of potential polyphenolic compound, catechins are naturally present in many fruits and plant-derived foods, especially in green tea [13]. These components constitute approximately 30–42% of the dry weight of the tea plant and can be classified into three categories: non-ester forms [catechin (C), epicatechin (EC), gallocatechin (GC), epigallocatechin (EGC)], ester forms [catechin gallate (CG), epicatechin gallate (ECG), gallocatechin gallate (GCG), epigallocatechin gallate (EGCG)], and other derivatives, such as catechin-3-O-gallate [14]. Numerous examples of evidence suggest that, like other flavonoids, catechins have a variety of bioactive properties that can improve animal health and prevent various diseases [15,16]. Meanwhile, catechins have good safety and compatibility when used in combination with other therapeutic agents such as vitamin C and tannic acid, which imply their potential as natural feed additives or supplements for animals, aimed at enhancing resilience and production performance [17,18]. Given the crucial role of the chicken farming industry in the global supply of meat and egg production, investigating the benefits, challenges, and limitations of catechins in chicken feeding provides a valuable theoretical framework. This paper focuses on the positive effects of catechins on intestinal health and the mechanism of maintaining productivity, providing an entry point for future research on the biological functions of plant-derived feed additives and their practical applications in the poultry industry.

## 2. The Chemical Structural Properties of Catechins

**Figure 1:**
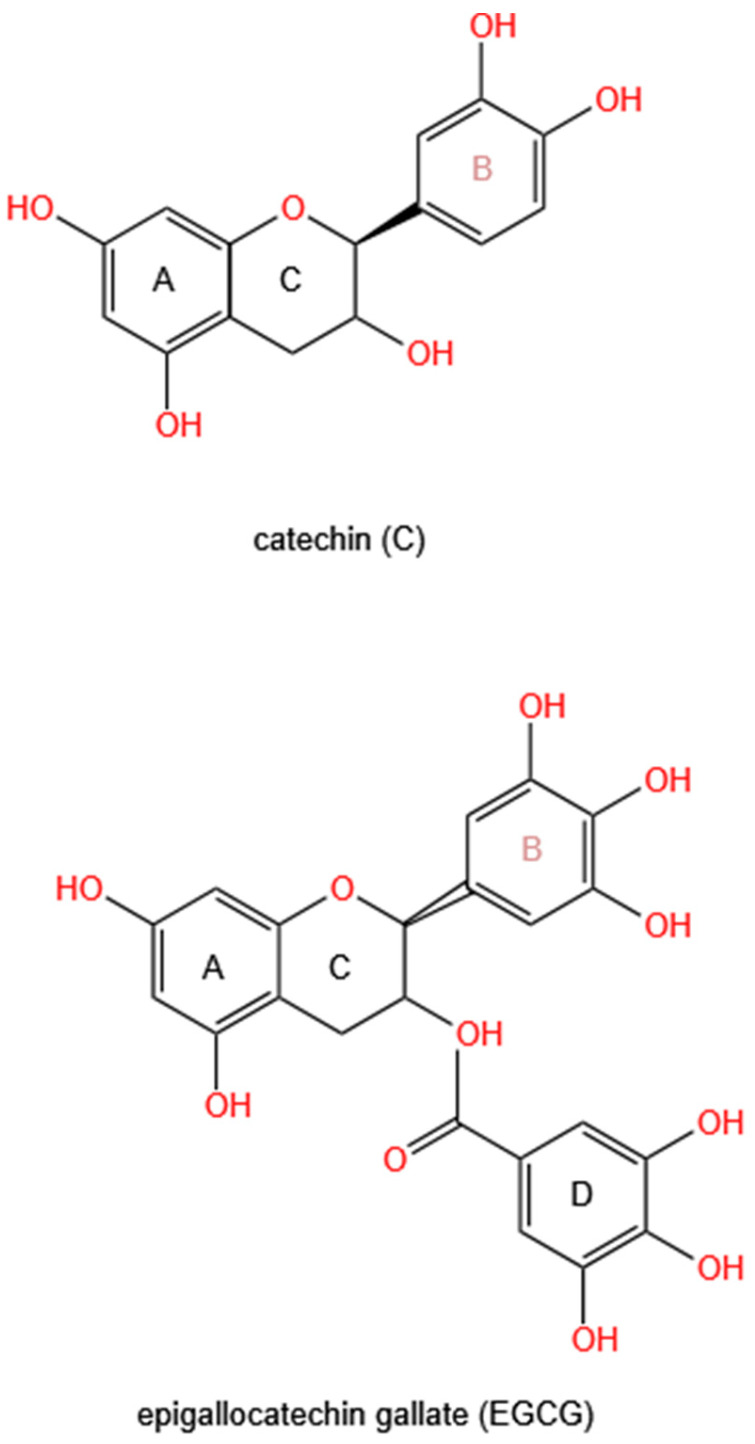
The chemical structures of two monomers of catechins. A: A ring; B: B ring; C: C ring; D: D ring.

Plant polyphenols, synthesized through metabolic pathways involving shikimic acid and phenylalanine, secondary metabolites in plants, are characterized by at least one phenolic hydroxyl group [19]. Categorically, they encompass tannins, flavonoids, and lignans, and the catechins specifically belong to the flavan-3-alcohols in flavonoids. Among the various catechins and their derivatives, EC, EGC, ECG, and EGCG constitute the major catechin monomer species present in green tea, collectively accounting for approximately 75% of tea polyphenols [20]. During the process of phytochemical processing or extraction, the structure of catechins may be influenced by factors such as temperature, pH, and other environmental conditions. However, catechins remain relatively stable under standard processing conditions (such as acidic environment, low temperature, light avoidance, and oxygen isolation) and exhibit low susceptibility to hydrolysis or oxidation [21]. The chemical formula of catechins varies by monomer type but generally consists of carbon, hydrogen, and oxygen atoms. Pure catechins typically form white to light yellow crystalline solids with high melting points and notable thermal stability [20]. The fundamental structure of catechins comprises A, B, and C rings. Additionally, some catechin derivatives, such as those esterified with gallic acid, feature a D ring as an auxiliary functional group on the flavonoid skeleton (Figure 1). These phenolic hydroxyl groups and benzene ring structures not only endow catechins with water solubility but also give them a certain degree of lipophilicity, ensuring that the phenolic hydroxyl groups in catechins molecules can exert a wide range of biological effects [22].

## 3. Catechins Promote Growth Performance and Product Quality

As an active ingredient originating from nature, catechins can exert a positive effect on animal production performance and product quality. For broilers, a previous study compared the effects of green tea by-products rich in catechin monomers at different levels (0.5% or 1% in diet) on the growth performance of red feather native chickens. The results showed that these by-products could increase feed conversion rate (FCR) by 13.5% to 14.7% and body weight (BW) by 23.8% to 52.0%, but had no effect on abdominal fat deposition [23]. However, Huang et al. reported that dietary supplementation of pure monomeric EGCG (40 or 80 mg/kg body weight per day) could significantly reduce the abdominal fat rate and expression of related pro-adipogenic genes in Ross 308 broiler chickens [24]. Additionally, Matsunaga et al. found that supplementing 1% loquat leaf by-products with low catechin content in diet had no effect on the body weight (BW) and feed intake (FI) of Tsushima Jidori crossbred chickens, but reduced muscle drip loss [25]. For laying hens, dietary supplementation of 200 mg/kg catechins did not affect the FCR and FI of Hy-Line Brown laying hens but could increase egg production by about 5% [26]. However, feeding high-tannin sorghum [5% catechin equivalents (CE)] had no effect on the percentage of hen-house production, egg weight, and egg-specific gravity of Isabrown pullet laying hens [27]. These studies indicate that the promoting effect of catechins on chicken growth performance and product quality may be related to the form and purity of catechins in additives and the species of chickens. Meanwhile, catechins also have a certain alleviating effect on the decline in chicken production performance caused by exposure to various adverse stimulations. For instance, previous studies proved that adding 300 or 600 mg/kg of EGCG into the basal diet remarkably alleviated the decreased BW and FI of AA broiler chickens caused by heat stress [28,29]. Wang et al. reported that the dietary supplementation of 130 mg EGCG/kg could confer protection against vanadium toxicity in laying hens, partially improving shell color and protoporphyrin IX [30]. However, it is worth noting that when catechins were used as food additives to treat chicken meat, it was found that compared with the control, the sensory color and meat color a* and b* values of chicken breast meat obviously decreased, indicating that catechins may not be suitable for food processing of chicken meat, but are more inclined to be used as feed additives to regulate chicken health [31].

## 4. The Antioxidant Property of Catechins

Oxygen-derived free radicals and related oxidants, including reactive oxygen species (ROS) and reactive nitrogen species (RNS), are ubiquitous, short-lived intermediates generated in aerobic organisms throughout their lifespan [32]. Under normal physiological conditions, low levels of these reactive species play essential roles in cellular functions by regulating oxidative modifications in biomolecules and key redox-sensitive signaling pathways. However, excessive oxidant formation leads to the accumulation of harmful byproducts, which can disrupt cellular function and structure, ultimately resulting in cell degeneration and death. Consequently, oxidative stress is linked to various pathological conditions, including intestinal dysfunction, chronic inflammation, reproductive disorders, muscular abnormalities, and impaired growth [33,34,35]. The production of cellular oxidants is counterbalanced by an antioxidant defense system, which includes low-molecular-weight antioxidants such as glutathione and ascorbic acid, reduced forms of renewable antioxidant enzymes, and ROS interacting enzymes such as superoxidative dismutase (SOD), catalase (CAT), and peroxidase [36]. This complex mechanism effectively protects cells from the harmful effects of oxygen free radicals and reduces the damage resulting from oxidative stress. In livestock production, oxidative stress is a critical concern as it can impair growth performance, reduce meat and egg quality, and increase susceptibility to diseases. Dietary supplementation with antioxidants has been shown to enhance the antioxidant capacity of animals, thereby improving overall health and productivity [37,38].

Notably, like many flavonoids extracted from plant tissues, catechins have been shown to exhibit potent antioxidant properties and function as effective ROS scavengers. It was found that dietary supplementation with catechins at different levels (50, 100, 150, 100, or 300 mg/kg) significantly reduced malondialdehyde (MDA) production, a marker of lipid oxidation, and enhanced oxidative stability in muscle, heart, and liver tissues of Cobb 500 broiler chickens [39]. The chemical structure of catechins and their derivatives contains multiple phenolic hydroxyl groups. These phenolic hydroxyl groups can directly undergo termination reactions with ROS and RNS by providing protons and electrons, thereby clearing or inhibiting the activity of free radicals [40]. Meanwhile, the adjacent hydroxyl groups in catechin molecules will partially dissociate, generating oxygen-negative ions that undergo complexation reactions with metal ions involved in the generation of oxygen-free radicals, reducing the catalytic effect of metal ions on oxidation reactions [41]. Therefore, the potential ability of catechins in scavenging excessive ROS and reducing oxidative stress follows the order: EGCG > ECG > EGC > EC > C [42,43,44]. Currently, among the catechin monomers, EGCG has been widely studied and utilized as a dietary additive in poultry production to prevent oxidative stress. Xue et al. demonstrated that the supplementation of EGCG (0, 300, and 600 mg/kg) significantly alleviated the decrease in antioxidant enzyme activities in broiler livers [29]. Furthermore, catechins can regulate pathways involved in antioxidant enzyme synthesis and signaling transduction, indirectly exerting antioxidant effects [40]. For instance, nuclear factor erythroid 2-related factor 2 (Nrf2) is a key regulatory factor in redox homeostasis. Nrf2 activity is positively regulated by the mitogen-activated protein kinase (MAPK) family member p38 or negatively inhibited by kelch-like ECH-associated protein 1 (Keap1). Upon activation, Nrf2 translocates to the nucleus, where it recognizes and binds to antioxidant response elements (ARE), thereby initiating antioxidant gene expression [45]. Previous studies have shown that by activating the Keap1/Nrf2 pathway or p38/Nrf2 pathway, dietary EGCG could promote the activities of antioxidant enzymes [such as SOD, CAT and glutathione S-transferase (GST)] in the eggs, lymphocytes, liver, kidney, and ovary of chickens [46,47,48]. However, the molecular mechanism of EGCG regulating pathways such as Keap1/Nrf2 has not been fully elucidated, and chicken breed, age, and health status may affect the antioxidant effect of EGCG.

## 5. Catechins Improve Intestinal Morphology and Alter Microbiota Composition

Healthy intestinal epithelial barrier function and optimal intestinal morphology are essential for animal growth and development, increasing immunity, resisting pathogens, and total health [49]. Key indicators of effective intestinal morphology involve elongated villus height (VH), shorter crypt depth (CD), and a higher ratio of villus height to crypt depth (VH/VD), which indicates well-developed enterocytes. The increase in VH not only enhances the contact area between the intestinal tract and chyme but also promotes the expression of nutrient transport proteins (such as sodium-glucose cotransporter 1 and aminopeptidase), which facilitates the absorption and utilization of nutrients [50]. Recently, increasing evidence confirms that catechins have a positive regulatory effect on intestinal health in chickens. For instance, catechin and its new ester derivatives (catechin pentaacetate and catechin pentabutyrate) injected into the amniotic membrane could significantly improve the intestinal morphology and alter the digestion and absorption capacity of Cornwall hybrid broiler chickens [12,51]. In addition, to maintain the integrity of the intestinal mucosal barrier and prevent villus atrophy and rupture, central components regulating intestinal homeostasis such as tight junctions (TJ), adhesive junctions (AJ), and desmosomes are intricately interconnected at the top part of intestinal epithelial cells [52]. These structures play a crucial role in preventing the invasion of pathogens and promoting the paracellular transportation of water, chemicals, and ions [53]. Protein families such as transmembrane proteins [occludin (OCLN), and claudins (CLDN)] and peripheral membrane proteins [zona occludens-1 (ZO-1)] are the fundamental components of TJ structures, while E-cadherin is integral to the AJ [52]. In the intestinal tract, oxidative stress induced by various sources disrupts the delicate balance of the cellular environment by impairing the integrity of tight junctions in the intestinal epithelium. Excessive ROS generation not only degrades tight junction proteins but also triggers inflammatory signaling pathways, further damaging intestinal health [54]. The promotive effects of catechins on intestinal growth in chickens are closely associated with their antioxidant properties. Studies have found that catechin-containing compounds can significantly enhance intestinal antioxidant capacity, inhibit the expression of interleukin 1β, and promote the expression of OCLN [12,51,55]. Meanwhile, Song et al. also reported that dietary EGCG could alleviate the gut oxidative injury and tight junction damage of heat stress-exposed broilers by increasing antioxidant capacity and inhibiting inflammatory response [56].

It is worth noting that catechins play a crucial role in modulating gut microbiota composition, indirectly affecting gut health [57]. The gut microbiota could modulate host gut health through various complex mechanisms, including nutrient metabolism, immune modulation, and antimicrobial functions, while an intact intestinal mucus layer and epithelial cells, in turn, facilitate the attachment and colonization, and provide nutrition or regulate their growth [58]. The ecological dynamics of avian gut microbiota, encompassing both harmful and beneficial microorganisms, are largely governed by interspecies competition for nutritional substrates and colonization niches in the gastrointestinal environment. Dominant bacterial phyla including *Firmicutes*, *Bacteroidetes*, *Proteobacteria*, *Tenericutes*, and *Actinobacteria* constitute fundamental components of poultry digestive systems, where they perform essential metabolic functions and mediate crucial biological regulatory mechanisms. This intricate microbial network features established inhabitants such as *Lactobacillus*, *Clostridium*, *Enterococcus*, and *Escherichia coli*, collectively demonstrating the multifaceted interactions within the avian digestive ecosystem [3]. *Lactobacillus* and *Bifidobacterium*, recognized as prominent probiotic strains, are capable of producing short-chain fatty acids (SCFAs) such as lactic acid and acetic acid through carbohydrate fermentation. These metabolites exert multiple beneficial effects on intestinal health, including promoting the proliferation of commensal microbiota while suppressing pathogenic colonization via pH reduction and competitive exclusion [59]. *Clostridium* (e.g., *Clostridium perfringens*) and *Campylobacter* (e.g., *Campylobacter jejuni*) are opportunistic pathogens linked to toxin-mediated diseases, and *Escherichia coli* exhibits strain-dependent duality: pathogenic variants cause infections, while commensal strains support microbial balance through metabolite production [60,61]. Catechins exhibit bacteriostatic activity against some pathogenic bacteria in chicken gastrointestinal tracts while demonstrating prebiotic potential through modulation of beneficial microbial proliferation. For instance, time–kill curve experiments in vitro proved that EGCG exhibited significant antibacterial activity against chicken-derived *Campylobacter jejuni* [62]. Meanwhile, recent studies in vivo have shown that catechin-enriched plant extracts and catechin derivatives significantly decrease the populations of *Lactobacillus*, *Clostridium*, and *Escherichia coli* [12,51,55,63]. Notably, catechin derivatives significantly promote *Bifidobacterium* proliferation, whereas plant extracts rich in catechins exhibit inhibitory effects on this genus. We speculate that different forms of catechins have a promoting or inhibiting effect on probiotics such as *Lactobacillus* and *Bifidobacterium* possibly due to synergistic interactions between catechins and other phytochemicals (e.g., tannins and flavonoids) in complex botanical matrices, which could alter microbial metabolic pathways or membrane permeability.

## 6. Conclusions and Perspectives

**Figure 2:**
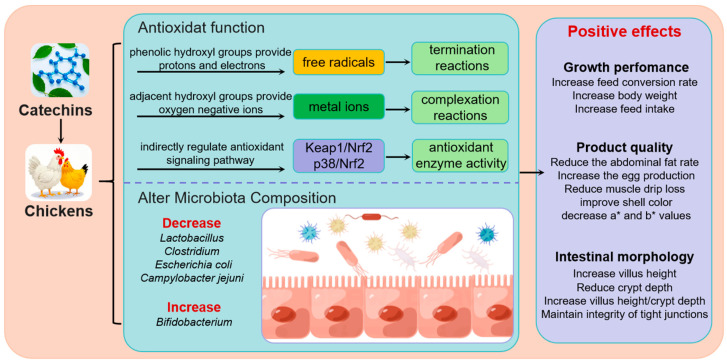
The mechanism of catechins improving chicken health. Keap1: kelch-like ECH-associated protein 1; Nrf2: nuclear factor erythroid 2-related factor 2.

Catechins, a group of polyphenolic compounds widely distributed in plants, have shown great potential as feed additives for regulating chicken intestinal health. This mini-review systematically summarizes the chemical properties of catechins, the mechanisms underlying their antioxidant functions, as well as their impacts on chicken intestinal health, gut microbiota, and production performance. By scavenging excessive ROS, catechins can effectively alleviate oxidative stress in the chicken tissues, protecting cells from oxidative damage. Additionally, catechins can inhibit the growth of harmful pathogens in the chicken intestine and promote the proliferation of beneficial bacteria, thus modulating the gut microbiota composition and maintaining the balance of the intestinal microecosystem. These positive effects on the intestinal environment ultimately contribute to improved chicken production performance, such as enhanced growth rate and feed conversion efficiency (Figure 2). In the context of the global trend towards reducing antibiotic use in animal husbandry, catechins have broad application prospects in poultry production. Future research should center on developing more efficient and stable catechin-based feed additives and exploring their synergies with other natural bioactive substances for chicken health. Meanwhile, long-term and large-scale in vivo studies across different chicken breeds and production settings are also essential to assess their safety and efficacy.

## Data Availability

No new data were created or analyzed in this study.
